# Exploiting the role of T cells in the pathogenesis of Sjögren’s syndrome for therapeutic treatment

**DOI:** 10.3389/fimmu.2022.995895

**Published:** 2022-10-28

**Authors:** Qi An, Jingwen Zhao, Xueqing Zhu, Baoqi Yang, Zewen Wu, Yazhen Su, Liyun Zhang, Ke Xu, Dan Ma

**Affiliations:** Third Hospital of Shanxi Medical University, Shanxi Bethune Hospital, Shanxi Academy of Medical Sciences, Tongji Shanxi Hospital, Taiyuan, China

**Keywords:** T cells, Sjögren’s syndrome, autoimmune, cytokines, T-cell treatment

## Abstract

Sjögrens syndrome (SS) is caused by autoantibodies that attack proprioceptive salivary and lacrimal gland tissues. Damage to the glands leads to dry mouth and eyes and affects multiple systems and organs. In severe cases, SS is life-threatening because it can lead to interstitial lung disease, renal insufficiency, and lymphoma. Histological examination of the labial minor salivary glands of patients with SS reveals focal lymphocyte aggregation of T and B cells. More studies have been conducted on the role of B cells in the pathogenesis of SS, whereas the role of T cells has only recently attracted the attention of researchers. This review focusses on the role of various populations of T cells in the pathogenesis of SS and the progress made in research to therapeutically targeting T cells for the treatment of patients with SS.

## Introduction

### T cell development, activation and differentiation

T cells originate from hematopoietic stem cells in the bone marrow and develop in the thymus, T cell receptor (TCR) diversity is generated through TCR gene rearrangement and then, T cells undergo positive selection to obtain major histocompatibility complex (MHC) restriction, expressing CD4 or CD8. The initial T cells then undergo negative selection to gain autoimmune tolerance. Gene mutations most likely occur in the variable region of the TCR and antigen recognition can only be achieved by binding to the MHC. If gene mutations occur in the TCR, negative and positive selection screening fails and some TCRs with different affinities to antigen peptides are preserved and differentiate into atypical and highly reactive T cells, which may cause autoimmune diseases (1–3).

The initial T cells enter the peripheral immune organs through the lymphatic and blood systems, and after TCR binds to the antigen (first signal) and co-stimulatory molecule (second signal), thereafter T cells become activated and differentiate into effector and memory T cells. The co-stimulator achieves the proinflammatory effect by activating T and B cells, whereas co-inhibitors inhibit activation. The immune checkpoint is mainly composed of co-stimulatory and co-inhibitory receptors or ligands. When external viruses and foreign bodies invade the body, immune cells become activated to produce an immune response, and immune checkpoints prevents excessive immunization (4).

T cells are divided into CD4^+^ and CD8^+^ T cells according to surface CD molecule expression. CD4^+^ T cells differentiate into specific subsets, depending on the presence of different cytokines, such as Th1, Th2, Th17, Treg, Tfh, and Tfr cells. CD8^+^ T cells differentiate into Tc1 and Tc2 cells and release interferon (IFN)-γ or IFN-α to regulate the immune response. Natural killer cell (NK)-like T cells directly kill cells and regulate immunity by secreting cytokines and chemokines that activate T and B cells. Memory T cells are mainly retained and can be quickly activated and differentiated into effector T cells when exposed to the same antigen: exerting an immune response. T cells play an important role in the occurrence and development of autoimmune diseases. This study reviews the role of T cells in the pathogenesis of SS and discusses SS therapies targeting T cells.

## Role of CD4^+^ T cells in SS

**Figure 1:**
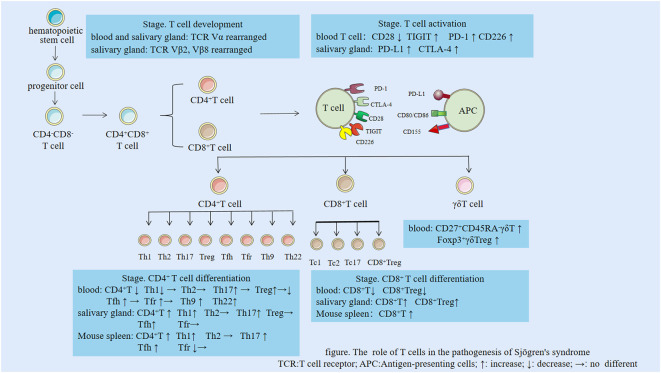
The role of T cells in the pathogenesis of Sjögren’s syndrome. TCR, T cell receptor; APC, Antigen-presenting cells; ↑: increase; ↓: decrease; →: no difference.

There are fewer CD4^+^ T cells in the peripheral blood of individuals with SS than in healthy individuals: mainly due to aging and decreased proliferative capacity of naive CD4^+^ T cells (5). Additionally, an increase in the number of CD4^+^ T cells was observed in the labial glands of patients in the early stage of SS. However, as the disease developed, the CD4^+^ T cell count in the labial glands gradually decreased, whereas the B cell count increased in the middle and late stages of SS (6). This increase in CD4^+^ T may be caused by CD4^+^ T cell migration from the peripheral blood to exocrine glands (7). In mouse SS models, the CD4^+^ T cell count increased in the spleen, lymph nodes, and exocrine glands, whereas the CD4^+^ T cell count in the peripheral blood has rarely been studied and the evidence is inconclusive (8). The following sections summarize the roles of different T cell subsets in SS (**Table 1** and **Figure 1** ).

**Table 1:** Role of CD4^+^ T cells in the pathogenesis of SS (pss:Primary Sjögren’s Syndrome).

Patients/Mouse	Sample	T cell subtype	effect	References
Patients(pss)	Peripheral blood	Th1,Th2	Th1 cell is significantly lower in patients with SS than that in healthy controls. No significant changes in Th2 cell. Decreased Th1/Th2. Th1 and Th2 cell are related to ESR and IgG levels.	(9, 10)
		Th1,Th2	Th1 and Th2 cell counts are not significantly different in patients with SS compared to those in the control group.	(11)
		Th17	Th17 cell count is significantly increased in patients with SS; no clear association between increased Th17 cell activity and disease symptoms.	(12–14)
		Th17	Th17 cell is not significantly different in patients with SS compared to that in the control group.	(11)
		Treg	Fewer CD25^+^ Tregs detected in patients with SS. CD25^+^ Tregs cell negatively correlates with ESR, CRP, IgG, and rheumatoid factor levels.	(15)
		Treg	CD25^+^FOXP3^+^ Tregs cell in patients with SS similar or higher than that in the healthy control group. CD25^+^FOXOP3^+^ Tregs cell is relatively low during the inactive stage of SS.	(16)
		Tfh	Tfh cell (CXCR5^+^PD-1^+^) is significantly higher in patients with SS compared to non-SS patients. Tfh cells stimulate B cell proliferation irrespective of disease activity.	(17, 18)
		Tfh	No changes in Tfh (CXCR5^+^PD-1^+^) cell in patients with SS compared with that in non-SS patients. Tfh cells are associated with disease activity and serum autoantibodies.	(19, 20)
		Tfh	Tfh (CCR7^low^PD-1^high^) cell in patients with SS is significantly increased compared to that in healthy controls and significantly correlates with disease activity scores and the number of differentiated B cells.	(21)
		Tfh,Tfr	Tfh17-like cells are significantly increased in patients with SS. Tfh17-like cells express PD-1, ICOS, CD40L, and IL-21 and could be involved in antibody-related immune response.	(22)
		Tfr	Tfr (FOXP3^+^) cell is significantly increased in patients with SS. The Tfr/Tfh ratio increase positively correlate with the degree of salivary gland infiltration. The Tfr cell correlates with the IgG4^+^ plasma cell count and the number, size, and irregularity of germinal centers.	(23, 24)
		Tfr	No notable changes in Tfr (FOXP3^+^) cell in patients with SS, except for increased Tfr cells in patients positive for autoantibodies than in patients negative for autoantibodies.	(24)
	Labial glands	Th1,Th2	local Th1 cell is increased; no significant change in Th2 cells and the Th1/Th2 is increased.	(25)
		Th17	Th17 cell is significantly increased in patients with SS compared to that in normal healthy control. No clear association between increased Th17 cell activity and disease symptoms. Th17 cells may contribute to disease progression.	(12–14)
		Treg	No notable changes in the CD25^+^ Tregs cell in bilateral parotid glands, parotid cells are not damaged by the increasingly abundant B and plasma cells. Salivary gland epithelial cells promote Tfh cell differentiation by producing IL-6 and expressing inducible T cell costimulatory ligand.	(15, 26, 27)
		Tfh	Tfh (CD4+CXCR5+) cell is increased in patients with SS compared to that in normal controls. Tfh cells facilitate B-cell differentiation, and their presence is associated with anti-nuclear antibodies	(28)
		Tfr	Tfr cell in patients with SS did not significantly change compared to that in healthy controls.	(24)
NOD Mouse	Peripheral blood	Th17	Flow cytometry showed increased frequency of Th17 cells in the peripheral blood of NOD mice. IL-17 promotes SS pathogenesis in an age-dependent manner.	(29)
	salivary gland	Th1,Th2	In a mouse model of SS with enlarged salivary glands: Th1 cell increased and Th2 cells either could not be detected or were present only in low amounts. IFN-γ induced CXCL10 and CXCL9 may play an important role in the destruction of lacrimal and salivary glands.	(30, 31)
		Th17	Flow cytometry showed increased frequency of Th17 cells in salivary glands of NOD mice.	(29)
		Tfh	Tfh (CD4^+^CXCR5^+^PD1^+^) cell was increased, IL-21 activated STAT3, and PAX and ID3 levels were decreased, resulting in an increase in the number of Tfh cells.	(32)
		Tfr	No changes in Tfr cells in the salivary glands of NOD mice.	(33)
	Mouse spleen	Th1,Th2	More Th1 cells in mice with SS than that in control; Th2 cells could not be detected.	(30, 31)
		Th17	Flow cytometry showed increased frequency of Th17 cells in NOD mice.	(29)
		Tfh	Tfh (CD4^+^CXCR5^+^PD1^+^) cell was elevated, but the Tfh1, Tfh2, and Tfh17 cell was the same as that in control mice.	(32)
		Tfr	No obvious change or reduction in Tfr (CXCR5^+^PD1^+^CD4^+^FOXP3^+^) cells. Tfr cells may limit the immune effect caused by a persistent antigen or infection.	(34)

### Th1 and Th2 cells

Changes in the number of Th1 and Th2 cells with disease progression were the focus of early research on SS. Immunohistochemical staining of salivary gland samples from patients with SS shows an increased number of local Th1 cells, but no significant change in Th2 cells, indicating an increased Th1/Th2 ratio (25). In contrast, the number of Th1 cells in the peripheral blood of patients with SS is significantly lower than that in healthy individuals, without significant changes in Th2 cells. Thus, in the peripheral blood, the Th1/Th2 ratio is decreased (9). This conclusion was confirmed in studies that used enzyme-linked immunosorbent assays to test the peripheral blood of patients with SS (10). Yet, Sudzius et al. found that the numbers of Th1 and Th2 cells in the peripheral blood of patients with SS were not significantly different from those in the control group (11). The observed opposite changes in the number of Th1 cells in the local salivary glands and peripheral blood may be explained by Th1 cell migration from the peripheral blood to glands, but there is no definitive correlation between the numbers of Th1 cells in these two locations. Some researchers believe that these changes reflect a systemic immunity reaction. Th1 cells mainly accumulate in the early stage of SS, before germinal center formation, whereas Th2 cells mainly appear during the infiltration stage of SS, after germinal center formation, when B cells in the salivary glands proliferate extensively. Therefore, it is possible that the studied patients were in the early-stage SS which explains the increase in the number of Th1 cells in the salivary glands and the lack of change in the number of Th2 cells (25, 35, 36).

Th1 and Th2 cells were also analyzed in the spleen and salivary glands of a mouse SS model. The spleen weight was increased because of the immune response. Enzyme-linked immunosorbent assays showed that Th1 cells in the spleen were increased, whereas Th2 cells were not detected. The number of Th1 cells was also increased in the enlarged mouse salivary glands, whereas Th2 cells were either undetectable or present only in small amounts. These findings might be explained by the generally small number of Th2 cells in mouse peripheral glands or the Th1/Th2 ratio imbalance; an increase in Th1 cells could inhibit Th2 cell differentiation (30, 31).

Th1 cells mainly secrete IFN-γ, interleukin (IL)-2, IFN-α, and C-X-C motif chemokine receptor 3 (CXCR3), of which INF-γ is the most important inflammatory factor (7). Early analysis of the saliva in patients with SS using the reference microsphere method that used probe labeling and immunohistochemistry showed that IFN-γ, TNF-α, and TNF-α/IL-4 levels were significantly increased, indicating that an increase in Th1 cell-related inflammatory factors may trigger SS (35, 37, 38). Interaction with IL-12, secreted by B cells and macrophages, prompts Th1 cells to induce an immune response. A small dose of IL-12 promoted CD4^+^ T cell differentiation into Th1 cells *via* the STAT4 phosphorylation pathway. IL-12 induces IFN-γ production, which stimulates IL-12 production, promoting Th1 cell differentiation. The lack of IL-6 normalizes IL-12 levels, alleviating SS manifestations in a mouse model (39). IL-27, a member of the IL-12 family, induces T, B, NK, and dendritic cell differentiation. Notably, IL-27 is elevated in the peripheral blood of patients with SS, which may promote Th1 cell differentiation *via* the STAT1 pathway, change the Th1/Treg cell ratio, and stimulate T cells to cause SS manifestations. IL-27 mediates inflammatory effects in autoimmune diseases such as SS and rheumatoid arthritis. Compared with normal non-obese diabetic (NOD) mice, IL-27-negative NOD mice had less inflammation in the lacrimal and salivary glands. Furthermore, T cells extracted from the spleen of NOD.*Il27ra* ^−/−^ mice and transferred to NOD. *Rag1* ^−/−^ mice failed to induce type 1 diabetes, which confirmed that the lack of IL-27 could prevent the occurrence of autoimmune diseases (40).

Th2 cells mainly secrete IL-4 and IL-13 and are involved in antibody formation and humoral immunity. Th2 cells are increased in the blood of patients with SS (41). Genotype investigation of cytokines regulating Th1/Th2 differentiation showed that their frequencies did not differ in disease activity between patients with SS and healthy controls (42). Thus, SS is primarily a Th1 cell-mediated condition, although Th2 cells may appear in mild cases (42). Mikulicz’s disease is a kind of IgG4-related diseases. Compared with primary SS, both diseases can cause lymphocyte infiltration in lacrimal gland and salivary gland. A comparison of patients with SS with a patient with Mikulicz’s disease showed that the two conditions had similar histological features in the lacrimal and salivary glands (43). The Mikulicz’s disease case was characterized by a prominent infiltration of IgG4-producing plasma cells, higher infiltration of IL-4 and IL-13, and lack of IFN-γ effect, whereas patients with SS had anti-SSA and anti-SSB antibodies, indicating that both conditions have a Th2 tendency. However, the sample size was too small to draw definitive conclusions (43).

### Th17 and Treg cells

Flow cytometry single cell analysis and immunohistochemistry revealed a significant increase in Th17 cells in the peripheral blood and labial gland tissues of patients with SS compared to that in normal healthy controls (12–14). However, one study found no such difference (11). The increased Th17 cell frequency was also noted in the peripheral blood, spleen, and salivary glands of NOD mice (29).

Th17 cells secrete IL-17A and participate in inflammatory responses in many autoimmune diseases. While IL-17A levels are significantly increased in the peripheral blood of patients with SS, it does not significantly correlate with an increase in disease activity or decrease after immunosuppressant treatment. However, immunosuppressant treatment significantly decreased IL-17 mRNA levels in patients with SS (44). The lack of significant differences in IL-17A levels in the peripheral blood of patients with SS and healthy controls may be due to the fact that IL-17A is produced by Th17 cells upon gland infiltration (45).

In a mouse SS model, IL-17A increased in the blood and salivary glands in a sex-depended manner. Inflammation in female mice occurs faster than in male mice, and the number of migrated lymphocytes is increased. Additionally, in the absence of IL-17, symptoms improve faster in female mice than in male mice (46).

Th17 cells exhibit strong plasticity and can be converted into Th1 cells. Th17 cells secrete IL-17A without producing IFN-γ. However, after stimulation with IL-12, Th17 cells start secreting IFN-γ. IL-17A^+^IFN-γ^+^ Th1 cells differ substantially from classical CD4^+^ Th1 cells in the submandibular and lymph nodes; therefore, Th1 cells were hypothesized to be transformed by Th17 cells (47). Th1 cell subtypes transformed from Th17 cells may be more pathogenic than normal Th17 cells (13, 48). Mice injected with IL-17A into their salivary glands gradually developed dryness and had increased levels of serum antibodies. IL-17A promotes IL-8 participation in neutrophil chemotaxis at the injured site, induces the formation of germinal centers, and activates B cells (49). *Il17a* ^−/−^ mice immunized with the salivary gland proteins did not show SS signs, however they rapidly developed SS upon implantation of Th17 cells generated *in vitro (* 50). Furthermore, the number of Th1 cells increased, indicating that Th17 cells infiltrated the gland *via* the Th1 pathway, which might be because of the plasticity of Th17 cells (50).

Regulatory T cells (Tregs) are immunomodulatory CD25^+^FOXP3^+^cells that suppress effector T cell activation. Fewer CD25^+^ Tregs were detected in the peripheral blood of patients with SS, whereas their number did not change in the bilateral parotid glands and the parotid gland cells were not damaged (15). The number of CD25^+^FOXP3^+^ Tregs in the peripheral blood of patients with SS was the same as or higher than that in the healthy control group, whereas during the inactive stage of SS, the number of CD25^+^FOXOP3^+^ Tregs was relatively low (16). Immunohistochemical staining showed no significant changes in CD25^+^ Tregs in the labial glands of patients with SS and normal individuals (15, 26, 27). However, the differences in these results may be related to the different surface markers used in the experiments (51).

T and B lymphocytes increase with an increase in Tregs upon ageing. However, Tregs lose their immunosuppressive function with age. Tregs are plastic, and are converted into Th1, Th2, and Th17-like cells to secrete related inflammatory factors. In NOD mice, the lacrimal gland inflammation and the number of Tregs increased with age, but no inhibition of Treg function was observed. Whether Tregs are converted to other cells with age cause inflammation, or become less inhibitory remains to be established (52).

Furthermore, sex is an important factor in SS. Estrogens promote Treg cell immunosuppression, reducing lacrimal gland inflammation, whereas androgens inhibit Treg cell function. Previous studies discovered that males with SS were primarily affected by lacrimal gland inflammation, whereas females with SS were affected by salivary gland inflammation. Lacrimal gland inflammation occurs with or without Treg cells in lymphocyte transfer in male NOD mice, which suggest that androgens prevent Treg cell function. Androgen’s involvement in the development of lacrimal gland inflammation is suspected as female NOD mice rarely trigger lacrimal gland inflammation, whereas lymphocyte transfer from female to male mice causes lacrimal gland inflammation (53).

### Tfh and Tfr cells

The median number of T follicular helper (Tfh) cells (CXCR5^+^PD-1^+^) in the peripheral blood of patients with SS is significantly higher than in individuals without SS, and Tfh cells stimulated proliferation of B cells unrelated to disease activity (17, 18). The frequency of Tfh cells in the peripheral blood of SS patients is not significantly increased compared to that in healthy controls, but there were more activated Tfh cells. Furthermore, the number of CCR7^low^PD-1^high^Tfh cells in the peripheral blood of patients with SS is significantly increased, indicating that CCR7^low^PD-1^high^Tfh cells may be involved in glandular inflammation and disease activity (21). There are many subtypes of Tfh cells, such as Tfh1, Tfh2, and Tfh17-like cells, the latter are significantly increased in the peripheral blood of patients with SS (22). Additionally, some experiments showed that CXCR5^+^PD-1^+^ Tfh cells in the blood of patients with SS were unchanged compared with those in individuals without SS, but the effects of Tfh1-like cells were more significant than those of Tfh2 and Tfh17-like cells (19, 20). An increase in Tfh (CD4^+^CXCR5^+^) cells was detected in the labial glands of patients with SS compared to that in normal controls. Furthermore, Tfh cells facilitated B-cell differentiation and their presence was associated with anti-nuclear antibodies (28).

In a mouse SS model, Tfh (CD4^+^CXCR5^+^PD1^+^) cells were elevated in the spleen and salivary glands, and there was no difference in the number of Tfh1, Tfh2, and Tfh17 cells in the spleen when compared to those in controls. IL-21 inhibited Tfh activation by spleen DNA binding inhibitor 3 (ID3) in NOD mice *in vivo*, whereas an IL-21 blocking agent injection in NOD mice decreased the number of Tfh cells and reduced the inflammatory reaction. ID3 attenuates mouse salivary and lacrimal gland inflammation and may act as part of the PAX3-ID3 signaling pathway, as IL-21R-Fc administration augmented the number of PAX3- and ID3-positive cells in the salivary glands of NOD mice, while reducing the pathologically increased proportion of Tfh and IL-17-producing T cells (32). Stimulation of this pathway may be beneficial for the treatment of SS and other autoimmune diseases (32). Germinal center enlargement in the spleen was also observed in a mouse SS model, and the number of Tfh (CD4^+^CXCR5^+^) cells positively correlated with disease status, confirming the importance of Tfh cells in SS (54).

Tfh cells expressing CXCR5, PD-1, and IL-21, interact with B and T cells and secrete related cytokines to promote germinal center formation. However, CCR9^+^ Tfh cells in the peripheral blood are more closely related to SS than CCR5^+^ Tfh cells. CCR5^+^ Tfh cells promote high CCL5 expression and cause inflammatory cell accumulation by secreting IL-7 (55, 56). Tfh cells can be present in the germinal centers in patients with late-stage SS, affecting the secretion of antibodies by B cells. Tfh cells in the peripheral circulation differ from Tfh cells in the germinal center in that they express little or no BCL-6. Circulating Tfh1 cells secrete IL-21, IFN-γ, and IL-10; Tfh2 cells secrete IL-21, IL-4, and IL-13; whereas Tfh17 cells secrete IL-17, IL-21, IL-22, and other cytokines (57, 58). Therefore, if different Tfh surface markers are selected in the peripheral blood and tissues, the expression of different populations of Tfh cells in SS may vary.

The number of T follicular regulatory (Tfr) (FOXP3^+^) cells was significantly increased in the peripheral blood of patients with SS, and the Tfr/Tfh ratio increase was positively correlated with the degree of salivary gland infiltration. The Tfr/Tfh ratio can also show the presence of ectopic lymphoid tissue (23, 24). No changes in the number of Tfr (FOXP3^+^) cells in the peripheral blood of patients with SS were observed in other studies. However, the number of Tfr cells in autoantibody-positive patients was higher than that in autoantibody-negative patients. Additionally, the number of Tfr cells in the labial gland of patients with SS did not significantly change compared to that in healthy controls (24). In a mouse SS model, no changes in the number of Tfr (CXCR5^+^PD1^+^CD4^+^FOXP3^+^) cells in the spleen were observed (34). Tfh and Tfr cells were found in the cervical lymph nodes of NOD mice, but no changes in the number of Tfr cells in the salivary glands were noted. Tfr cells share the same CXCR5 recognition signal as Tfh cells, and express FOXP3 and PRDM (33). The FOXP3, PD-1, and ICOS expression levels in Tfr cells from tissues and blood vary greatly. There are more Tfr cells in peripheral blood than in tissues, indicating that Tfr does not completely inhibit B cells and enters the blood after secondary lymphoid tissue production and before the action of B cells (59, 60).

### Th9 cells

Th9 cells, first discovered in 2008, are a new subtype of CD4^+^ T cells. TGF-β and IL-4 induce differentiation of CD4^+^ T cells into Th9 cells, which secrete IL-9. Th2 cells can be converted into Th9 cells, which are induced only by TGF-β. Additionally, IL-21, IL-10, TNF-α, and TLR2 promote Th9 cell differentiation, whereas IFN-γ and PRDM inhibit their differentiation (61). IL-9 inhibits Tregs, promotes Th17 cell differentiation, and participates in B-lymphocytic tumor development (62, 63). Th9 cells are significantly elevated in the peripheral blood and synovial tissue of patients with RA compared to that in healthy controls (64). There was no significant decrease in Th9 cells in the peripheral blood after infliximab treatment (65). Similarly, the proportion of Th9 cells in the peripheral blood of patients with systemic lupus erythematosus was also increased. The number of Th9 cells in the spleen and kidneys of lupus mice was higher than that in normal mice and correlated with germinal center formation (66). Flow cytometry showed an increased number of Th9 cells and no significant changes in IL-9-producing Th2 cells in the peripheral blood of patients with systemic sclerosis (67). Although there has been no relevant research on Th9 cells in SS, the above-mentioned evidence indicates the importance of Th9 cells in various autoimmune diseases.

### Th22 cells

In addition to secreting IL-22, IL-13, and TNF-α, Th22 can be converted into Th1 and Th2 cells under certain conditions. IL-6 and TNF-α promote Th22 differentiation, whereas high doses of TGF-β inhibit Th22 cell differentiation (68). A peripheral blood flow assay using CCR4^+^, CCR6^+^, and CCR10^+^ as Th22 cell surface markers showed that Th22 cells of patients with RA were significantly increased compared with that in the healthy control group; after methotrexate and leflunomide treatment, the peripheral Th22 cell levels were decreased in patients with RA (69). Additionally, both IL-22 levels and Th22 cell counts were significantly higher in the peripheral blood of patients with SLE than that in the normal controls and correlated with disease activity (70, 71). However, one trial found lower peripheral blood IL-22 and higher IL-17 and IL-23 levels in patients with SLE compared to that in healthy controls, suggesting independence to disease activity. This could possibly be because of the specific subset of IL-22-producing CD4^+^ T cells in human peripheral blood (72). In a study where the salivary glands of 19 patients with primary SS and 16 patients with non-specific sialadenitis were biopsied by immunohistochemistry, IL-22 expression of patients with SS and the control group was increased (73). The comparison between SS patients and the healthy control group showed that the serum IL-22 level of SS patients was significantly increased, and the serum IL-22 level was significantly positively correlated with anti-SSA, anti-SSB, and rheumatoid factor (RF) (74). Although studies on Th22 in SS are lacking, Th22 cells have shown a relative advantage in various autoimmune diseases.

The comparison between SS patients and the healthy control group showed that the serum IL-22 level of SS patients was significantly increased, and the serum IL-22 level was significantly positively correlated with anti-SSA, anti-SSB, and RF. Although studies on Th22 in sjogren’s syndrome are lacking, Th22 cells have shown a relative advantage in various autoimmune diseases.

## Role of CD8^+^ T lymphocytes in SS

The decreased number of CD8^+^ T cells in the peripheral blood of patients with SS may be due to persistent viral infection. CD8^+^ T cells secrete perforin and kill cells infected by viruses. Therefore, the participation in the elimination of viral infections continuously depletes CD8^+^ T cells from the peripheral blood (75). The decrease in circulating CD8^+^ T cells may also be due to their migration to and accumulation in the glands (76). CD8^+^ T cells accumulate in the exocrine glands of patients with SS. CD8^+^ T cells can differentiate into Tc1 cells that secrete IFN-γ and TNF-α and have strong killing activity. They can also differentiate into Tc2 cells that secrete IL-4 and IL-13 that enhance inflammation. Tc17 cells secrete IL-17, and CD8^+^ Tregs secrete IL-21 (76). CD8^+^ Tregs, that inhibit lymphocyte function and prevent the continuous development of immune responses, are significantly reduced in the peripheral blood of patients with SS. In contrast, in the late stage of SS, the number of CD8^+^ Tregs may increase (77). Generally, the number of CD8^+^ Tregs cells are undetectable or slightly reduced in the peripheral blood of SS, suggesting that it is inversely proportional to disease activity, and there are also large numbers of IFN-γ and CD8^+^ Tregs in the lacrimal gland, that inactivate dendritic cells and thereby alleviate SS manifestations (76). In NOD mice with CD8^+^ Treg depletion, the activation of dendritic cells and stimulation of the generation of CD4^+^ T cells that secrete IL-17A increased the number of Th17 cells, leading to corneal damage and more severe SS pathology. A decrease in CD8^+^ Treg cells and an increase in Th17 cells are important factors involved in SS aggravation (78). The CD4^+^CD8^+^ T cells were significantly increased in the peripheral blood of patients with SS compared to that in healthy controls. The number of these cells positively correlated with IL-10 levels and negatively correlated with disease activity, possibly indicating a protective role of CD4^+^CD8^+^ T cells in SS (79, 80). CD8^+^ T cells in the labial glands of patients with SS are excessively active and characterized by abnormal proliferation, therefore the tissue accumulates memory CD8^+^ T cells, which secrete high levels of IFN-γ, leading to SS manifestations (76). Immunofluorescence detection experiments also revealed an increased number of tissue-resident memory CD8^+^ T cells in the salivary gland epithelial cells of patients with SS (81).

CD8^+^ T cells were increased in both the spleen and salivary glands of NOD mice (78, 81), especially in the late stage of SS. CD8^+^ T cells reside in glandular tissues and cause apoptosis of salivary gland cells through the FASL and CTL pathways. Knockout of IFN-γ in mice alleviated salivary acinus atrophy, restored the secretory function, and reduced the germinal center size (81). It is possible that IFN-γ released by CD8^+^ T cells in the submandibular glands and acting on CXCR3 caused the destruction of the acinus. The same study also found that depletion of CD8^+^ T cells normalized the antibody secretion of salivary glands and blood levels in mice. CD8^+^ T cells could be used as a target to treat SS (81)(**Table 2**).

**Table 2:** Role of CD8^+^ T cells in the pathogenesis of SS.

Patients/Mouse	Sample	T cell subtype	effect	References
patients(pss)	Peripheral blood	CD8^+^T cell	The expression of CD8^+^ T cells in patients with SS is reduced. Latent viral infection in SS may persistently trigger a decrease in cytotoxic CD8^+^ T cells and maintain clonal expansion.	(75, 76)
		CD8^+^Treg	CD8^+^ Tregs are significantly reduced in patients with SS, however in the late stage of SS, the number of CD8^+^ Tregs cells tends to increase, which inhibits the activation of dendritic cells and reduces the number of CD4^+^ Tfh cells.	(76, 77)
		CD4^+^CD8^+^T cell	The number of CD4^+^CD8^+^ T cells in patients with SS was significantly increased compared to that in healthy controls, positively correlated with IL-10 levels, and negatively correlated with disease activity.	(79, 80)
	Labial gland	CD8^+^T cell	CD8^+^ T cells in patients with SS, especially tissue-resident memory CD8^+^ T cells that secrete high levels of IFN-γ, were shown to be hyperactive and proliferate abnormally. CD8^+^ T cells induce apoptosis and lysis of acinar epithelial cells in patients with SS through the Fas/FasL pathway.	(76, 81)
NOD Mouse	salivary gland	CD8^+^T cell	The number of CD8^+^ T cells in NOD mice was increased, especially in advanced SS. IFN-γ produced by CD8^+^ T cells in NOD mice destroyed the integrity and function of tight junctions in salivary gland epithelial cells and mediated the death of salivary gland epithelial cells.	(78, 81)
	spleen	CD8^+^T cell	The number of CD8^+^ T cells was increased in NOD mice, especially in advanced SS.	(78, 81)

## Role of NK-like cells in SS

Natural killer (NK) cells are natural immune cells. The total number of NK cells in the peripheral blood of patients with SS is decreased compared to that in healthy controls. NK cell activity and expression levels of the NK cell activation receptors CD2, NKG2D (C-type lectin-like NK receptor), and NKP62 (natural cytotoxic receptor), as well as serum IL-18 and TNF-α levels are elevated in patients with SS (82–85). The proportion of CD56^+^ NK cells in patients with SS was also found to be higher than in healthy controls. The ratio of CD56 strongly positive cells to CD56 weakly positive cells positively correlated with IgG levels and was directly proportional to disease activity, indicating that this ratio could be used as a specific indicator of SS. In healthy people, the number of CD56^dim^ cells is 10 times higher than that in CD56^bright^ cells, whereas in SS, the number of CD56^bright^ cells increases, while the number of CD56^dim^ cells decreases, leading to an overall increase in the CD56^bright^/CD56^dim^ ratio. Secreted cytokines tend to migrate to the glands, causing inflammation. Upon the treatment of patients with SS with hormones and immunosuppressants, the number of CD56^dim^ NK cells increased, but the CD56^bright^/CD56^dim^ ratio and number of CD56^bright^ NK cells did not change (86). NKP30 is an NK cell-specific activation receptor that regulates the interaction between NK and dendritic cells and stimulates IFN-γ secretion to promote salivary gland inflammation. The number of NKP30^+^ (CD56^+^CD16^+^) NK cells in the peripheral blood of patients with SS is increased, and this parameter is sensitive to formal immunotherapy (87, 88).

Additionally, there is another type of NK-like T cells, which is divided into type I NK-like T cells (iNKT) and type II NK-like T cells. iNKT cells, which produce IL-17 and IFN-γ, are elevated in the peripheral blood of patients with SS, but are not detected in the labial glands, possibly because they are difficult to identify in the labial glands using the existing technology (89, 90). NK-like T cells have the same functions as both CD3^+^ T and CD56^+^ NK cells. The number of NK-like T cells in the peripheral blood of SS patients was decreased compared to that in the healthy group, which positively correlated with the numbers of CD4^+^ T, CD8^+^ T, and NK cells, but not with that of B cells (91, 92). As SS developed, NK-like T cells gradually decreased, and the number of NK-like T cells in treated patients gradually returned to normal (93). In other studies, the number of NK-like T cells in the peripheral blood of SS patients was increased, which may be due to insufficient sample size, or different course of disease and treatment between patients (94–96). It has also been reported that the numbers of CD3^+^CD161^+^ NK-like T cells in the peripheral blood of patients with SS and healthy controls were not significantly different. This discrepancy may be due to the detection of different surface markers of NK-like T-cells (82). The numbers of NK cells in the labial glands of patients with SS and salivary glands of NOD mice were increased compared to the respective numbers in unaffected individuals, presumably due to the migration of NK cells from the peripheral blood to the salivary gland tissue (97).

## Role of memory γδT cells in SS

γδT cells, a type of T cell present in the skin, colon, and reproductive tract, with a limited number detected in peripheral blood and lymphocytes, play specific biological roles. γδT cells are separated into CD4^+^ γδT, CD8^+^ γδT, and γδTreg cells. CD4^+^γδT cells express Th1-, Th2-, and Th17-related cytokines, CD8^+^γδT cells are primarily connected with viral infections, and γδTreg cells’ physiological activities are still being researched (98). Human peripheral blood γδT cells can be classified according to their expression of CD27 and CD45 as follows: naive (CD27 ^+^ CD45 ^+^), effector memory (CD27^+^CD45^-^), central memory (CD27^+^CD45^-^), and terminally differentiated (CD27^-^CD45^+^), patients with SLE have more CD27^+^CD45^-^γδT cells in their peripheral blood and fewer Foxp3^+^γδTreg cells that express Foxp3 compared to healthy controls, as well as a substantial increase in Vδ1 cells after glucocorticoid and cyclophosphamide treatment (99). After treatment with biologic agents, CCR6^+^ Vγ9^+^Vδ2^+^ T cell counts were significantly reduced and returned to normal in the peripheral blood of patients with psoriasis compared to healthy controls (100). Therefore, γδT cells play an important role in autoimmune diseases, but the function of γδT cells in dry syndrome still needs further investigation.

## Role of memory T cells in SS

CD4^+^ memory T cells were increased in the bilateral parotid glands of patients with SS compared to healthy controls, whereas there were fewer CD8^+^ memory T cells. However, both cell types could lead to glandular fibrosis. The CD4^+^CD45^+^ memory T cells in the parotid gland are very similar to Tfh signals that help B cell recruitment and form germinal centers, therefore induce SS (101, 102). Large numbers of tissue-resident CD8^+^ and CD4^+^ memory T cells were detected in patients and in a mouse SS model: the number of CD8^+^ memory T cells was greater than that of CD4^+^ memory T cells, and CD69^+^CD103^+^ memory CD8^+^ T cells were predominant. When these CD8^+^ memory T cells were ablated in mouse experiments, the pathological manifestations of SS in mouse salivary glands were alleviated (81).

## Role of T cell checkpoint in the pathogenesis of SS

T-cell checkpoints, which include co-stimulatory and co-inhibitory signals, are secondary signals for T-cell activation. T-cell checkpoint receptor dysfunction is associated with autoimmune disease development. CD28, PD-1, CTLA-4, CD226, and TIGIT are expressed on T cells and CD80/CD86, PD-L1, and CD155 are expressed on antigen-presenting cells. CD28, CD226 are co-stimulatory signals; CD28 binds to CD80/CD86 and CD226 binds to CD155, decreasing the TCR threshold and promoting T cell proliferation, survival, and cytokine production. CTLA-4, PD-1, TIGIT are co-inhibitory signals; CTLA-4 binds to CD80/CD86, PD-1 to PD-L1, and TIGIT to CD155, inducing cell survival, preventing apoptosis, and downregulating or terminating T cell activation (103).

Soluble CD28 expression was elevated in the peripheral blood of patients with SS with deficiency of T cells. Whereas CTLA-4 was elevated in the salivary glands of patients with SS, which was associated with disease susceptibility and the presence of autoantibodies (104–106). PD-1 and its ligand PD-L1 play an important role in autoimmune diseases. PD-1 is expressed on T cells, and its interaction with PD-L1 inhibits TCR signal transduction and induces immature CD4^+^ T cells differentiation into Tregs, thereby suppressing immune reactions. PD-1 expression on T lymphocytes in the peripheral blood of patients with SS was increased compared to that in healthy controls, but PD-1 did not correlate with the levels of anti-nuclear antibodies, immunoglobulins, or the erythrocyte sedimentation rate. Patients with SS have higher PD-L1 expression in the salivary gland epithelium, likely because of stimulation by IFN-γ. PD-1/PD-L1 expression changes may contribute to the pathogenesis of SS. The role of the PD-1/PD-L1 interaction in SS has not been thoroughly investigated and further research is required (107, 108). Recently, it was discovered that CD226 and TIGIT proteins, which are competitively expressed on T and NK cells as novel immune checkpoint proteins, were significantly elevated in the peripheral blood of patients with SS. Further, TIGIT^+^CD4^+^ T cells in patients with SS showed greater activity than those in healthy individuals. CD226 is a co-stimulatory molecule, whereas TIGIT is a co-inhibitory molecule that binds to its ligand CD155, competing with CD226, to exert its inhibitory function. The disrupted balance between the co-stimulatory and co-inhibitory molecules is an important factor that causes an immune response. Anti-CD226 and anti-TIGIT treatments maintain the immune balance by regulating Tregs, which is important for rheumatic disease treatment (109). Notably, dryness symptoms caused by the treatment of tumors with immune checkpoint inhibitors are different from those caused by SS. The former mainly occur in older males, tend to have less ocular involvement, and are accompanied by different antibody and histopathological features (110, 111). In conclusion, the application of modulator immune checkpoints as novel therapeutic targets for the treatment of SS remains to be investigated.

## Role of TCR in the pathogenesis of SS

TCR is composed of an α-chain and a β-chain, and each chain includes a variable, easily mutated region, and a constant (C) region. In patients with SS and in mouse models of SS, the TCR is rearranged. The variable region of TCR in T cells from salivary gland cells in patients with SS does not play a corresponding role in inflammation, and the preferential amplification of Vβ transcripts, especially Vβ2 and Vβ8, is likely to be closely related to the stimulated immunity (112). Vα was also found to be rearranged in T cells from salivary glands, and antigen-specific and clonal expansion of T cells in the peripheral blood and salivary glands of patients with SS was studied using single-cell analysis. The salivary gland expansion clones and TCRα sequence frequencies were increased in patients, and salivary glands became hypofunctional. The TCRα sequence starts simultaneously in two gene sequences, which may lead to increased levels of gene expression and elevated self-reactivity. These phenomena may provide an important basis for SS development (113, 114). The expression levels of the first 50 differential genes in the minor salivary glands of patients with primary SS and normal control groups were compared by quantitative reverse transcription polymerase chain reaction, and it was found that TCR played an important role in T lymphocyte activation (115). Peripheral blood samples from patients with primary SS and healthy control groups were studied using single-cell RNA sequencing technology and TCR variable genes CD4^+^ T cells were found to be amplified in patients with primary SS (116). Single-cell RNA sequencing analysis of the submandibular glands of mice with Sjogren’s syndrome revealed that TCR signals were enriched in the mice with SS (117).

## Development of T cell therapy in SS

**Figure 2:**
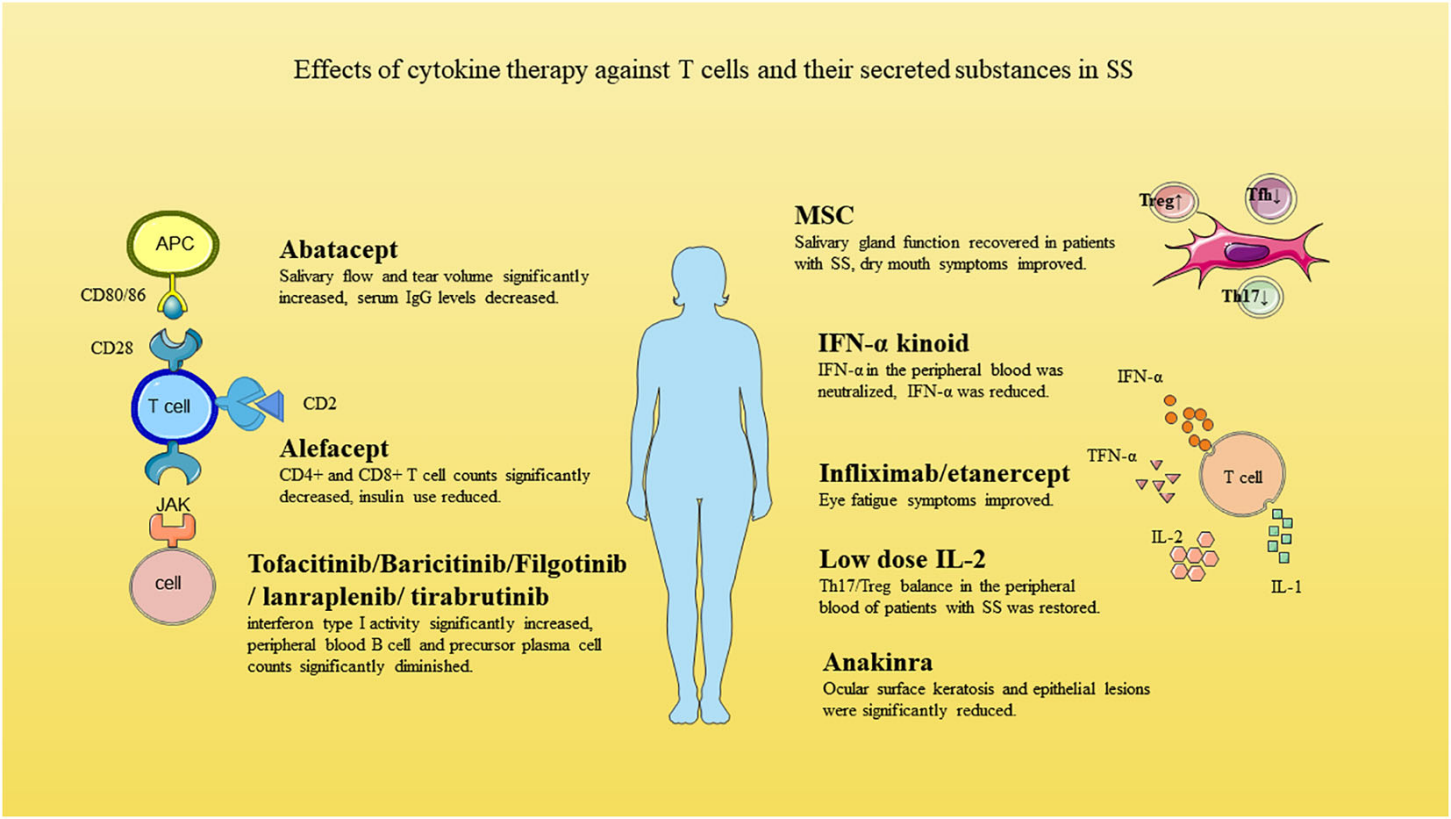
Effects of cytokine therapy against T cells and their secreted substances in SS.

There are no well-established treatments targeting T cells and their secreted inflammatory factors in SS, but it is an actively researched topic (**Table 3** and **Figure 2**).

**Table 3:** Effects of cytokine therapy against T cells and their secreted substances in SS.

Drugs	Target	Drug effects	References
Abatacept	Blocking CD80/CD86 and CD28	Salivary flow and tear volume significantly increased, serum IgG levels decreased, and drying symptoms improved.	(118)
		Glandular manifestations of SS were not significantly improved.	(119)
Tofacitinib	JAK1 and JAK3 inhibitor	Dry eye symptoms improved.	(120)
Baricitinib	JAK1 and JAK2 inhibitor	Arthritis and rash symptoms in patients with SS improved. Notable effects in patients with the active stage of interstitial lung disease.	(121)
Filgotinib, lanraplenib, tirabrutinib	JAK-1, SYK, BTK inhibitors	RF, IgM, IgG, and IgA levels decreased, interferon type I activity significantly increased, peripheral blood B cell and precursor plasma cell counts significantly diminished, and ESSDAI score was decreased.	(122)
Alefacept	Anti-CD2 dimer fusion protein	CD4^+^ and CD8^+^ T cell counts significantly decreased, insulin use reduced, and hypoglycemia was attenuated.	(123)
MSC	Inducing Treg cell growth in CD4^+^ T cells	Salivary gland function recovered in patients with SS, dry mouth symptoms improved, and the inflammatory response was inhibited.	(121, 124, 125)
IFN-α kinoid	Anti-IFN-α vaccine	IFN-α in the peripheral blood was neutralized, IFN-α was reduced, and dry mouth symptoms were improved.	(126)
Infliximab/etanercept	TFN-α	Eye fatigue symptoms improved, but treatment was ineffective for dry eyes, dry mouth, and SS.	(127)/ (128)
Tocilizumab	Recombinant humanized monoclonal antibody against human IL-6 receptor	Systemic involvement and dryness in SS were not improved.	(129)
Low dose IL-2	Tregs	Th17/Treg balance in the peripheral blood of patients with SS was restored.	(130)
Anakinra	IL-1 receptor antagonist	Ocular surface keratosis and epithelial lesions were significantly reduced, and ocular surface fatigue was alleviated.	(131)

Abatacept inhibits T cell proliferation by blocking the co-stimulatory signals of CD86/CD80 on antigen-presenting cells and CD28 on T cells, thereby inhibiting the immune response, reducing inflammation, and increasing the secretion of the salivary glands. Tfh cells are also reduced by this treatment. In a 1-year multicenter study of 68 patients with SS treated with intravenous abatacept, saliva flow and tear volume were significantly increased, serum IgG levels were decreased, and dry symptoms were improved (118). The effect was more significant at early treatment intervention. In 15 patients, adverse reactions, mainly infections, were observed and most of these were transient (118). In another randomized, double-blind, controlled trial of 187 patients with SS, abatacept treatment did not significantly improve gland characteristics in individuals with SS. Twenty patients experienced severe adverse reactions, such as septic shock and drug hypersensitivity. Current clinical trials are single-center phase III trials (119).

Applying Tofacitinib application, an inhibitor of Janus kinase (JAK) 1/3, to dry eyes alleviated ocular inflammation and was well tolerated, so the drug is currently in phase I/II of clinical trials (120). Baricitinib, a selective JAK1 and JAK2 inhibitor, is used to treat RA. In a recently published clinical trial involving 11 patients with SS, baricitinib improved arthritis and rash symptoms, and significantly alleviated the active stage of pulmonary interstitial disease. It is currently in the initial stages of clinical trials (121). JAK 1 inhibitors, spleen tyrosine kinase, and selective Bruton tyrosine kinase filgotinib, lanraplenib, and irabrutinib, respectively, block corresponding signal transduction pathways, which might be beneficial for the treatment of SS. In a multicenter, double-blind, randomized study of 150 patients with primary or secondary SS treated with filgotinib, lanraplenib, tirabrutinib, and placebo, filgotinib-treated patients had reduced type I IFN activity, increased number of memory B cells, and decreased levels of RF, IgM, IgG, and IgA, respectively, compared with that in patients that received the placebo. IFN activity was significantly increased in the lanraplenib group, and the numbers of B cells and precursor plasma cells in the peripheral blood of patients treated with tirabrutinib were significantly decreased, as was the ESSDAI score (122).

Alefacept, originally developed for psoriasis, inhibits T cell activation and proliferation by binding to CD2 expressed on the T cell surface, causing a decrease in the numbers of CD4^+^ and CD8^+^ T cells. To the best of our knowledge, there has been no relevant research on the treatment of SS with alefacept. However, this drug was used in type I diabetes clinical trials, and treatment reduced insulin use and decreased the frequency of hypoglycemic events (123). It is currently in phase II of clinical trials for diabetes (132).

Mesenchymal stem cells (MSCs) that can be isolated from the bone marrow, fat, umbilical cord, and gums are characterized by their self-renewal ability and differentiation potential. MSCs also play an anti-inflammatory and immunomodulatory role. IL-10 induces CD4^+^ T cells to differentiate into Tregs, thereby weakening the immune response and alleviating SS (133, 134). Umbilical cord mesenchymal stem cells (UCMSCs) inhibit T cell proliferation in patients with SS, increasing the number of Tregs and lowering the number of Th17 and Th1 cells. In a clinical experiment involving 24 patients with SS, intravenous injections of UCMSCs led to the recovery of salivary gland function, improvement of dry mouth symptoms, and suppression of inflammatory reactions. No adverse reactions were observed and this treatment is currently in the final stage of a phase II trial (121, 124, 125).

The M3 acetylcholine receptor (M3R) is a G protein-coupled receptor that plays an important role in the secretory function of salivary and lacrimal glands. Autoantibodies against M3R and an increase in M3R reactive CD4^+^ T cells were detected in the peripheral blood of patients with SS. In an experimental mouse SS model, the M3R-reactive CD4^+^ T cells secreted IL-17 and IFN-γ. M3R-reactive CD4^+^ T cell peptide ligands inhibited IFN-γ production *in vitro* and alleviated salivary gland inflammation *in vivo*, thus the M3R-reactive CD4^+^ T cells may become a potential target for the treatment of SS (135). Other biological therapies targeting T cells, such as anti-CD40 and anti-ICOSL, are still under investigation.

## Targeting cytokines in SS

IFN-α kinoid is an anti-IFN-α vaccine that stimulates generation of an anti-IFN-α antibody in the peripheral blood of MRL/lpr mice. The reduced level of IFN-α improved dry symptoms of SS without adverse reactions. This treatment is still at the preclinical stage, and it requires many tests to assess its efficacy and suitability for clinical application (126). Interestingly, oral mucosal administration of a low IFN-α dose to 497 patients with SS in a phase III clinical trial significantly increased saliva flow without causing significant adverse events (136).

Infliximab and etanercept, which both target TNF-α, have been shown to improve ocular fatigue symptoms, but were ineffective in patients with dry eyes and dry mouth. Currently, a multicenter phase II clinical trial of infliximab is underway, but so far, xerosis symptoms and the degree of salivary gland infiltration were not improved and there were serious adverse reactions, such as transfusion reaction, autoimmune liver disease, and pneumococcal septicemia (127). In a clinical trial of subcutaneously applied etanercept in 15 SS patients, the salivary and lacrimal gland functions were not significantly improved, and some individuals had local side effects (128).

IL-6 is involved in most autoimmune reactions, and elevated IL-6 levels are observed in the peripheral blood and salivary glands of patients with SS. Therefore, IL-6-targeting treatment for SS may be promising. The recombinant humanized anti-human IL-6 receptor monoclonal antibody tocilizumab did not improve the systemic involvement and dry symptoms in a cohort of 55 patients with SS, compared with the manifestations in the placebo group (129). The treatment group is currently involved in a stage II/III clinical trial.

Low IL-2 doses increase the number of CD4^+^ Tregs and normalize the Th17 cell/Treg ratio, suppressing immune reactions. Administration of a low IL-2 dose to a cohort of 190 patients with SS restored the number of Tregs in the peripheral blood, which was significantly lower in patients, whereas the proportion of Th17 cells was not significantly changed in comparison to the healthy control group. Thus, therapy with low-dose IL-2 can restore the Th17/Treg balance in the peripheral blood of patients with primary SS, and this treatment has now completed its phase II clinical trial (130).

Blocking IL-7 and CXCR3 reduces the secretion of various cytokines by T cells. Compared to the control group, patients with SS had increased serum IL-7R levels and elevated IL-7 and IL-7R expression in the labial glands, and IL-7 was related to increased inflammation and lymphoma (137). Therefore, targeting the IL-7/IL-7R pathway may be an effective therapeutic strategy. IL-1 levels were significantly increased in the salivary glands of patients with SS, and this cytokine is an important target for autoimmune diseases. In IL-1 knockout autoimmune deficient mice, although there was no reduction in lymphocyte infiltration of the lacrimal gland or eye, ocular surface keratosis and epithelial lesions were significantly reduced, implying that the IL-1 receptor antagonist anakinra could be used for the treatment of ocular surface fatigue (131). IL-22 was significantly increased in the salivary glands and serum of patients with SS, indicating that IL-22 may also be an important therapeutic target for SS (138).

## Conclusion

Various T-cell subsets are essential for the development of SS. However, there are many T cell classifications and subtypes, and further research is needed to elucidate their mechanisms of action. This review summarized the current understanding of the roles of various T cell subsets in SS and discussed attempts to therapeutically target T cells. We hope that our review will be a useful primer for everyone interested in the development of SS therapies.

## Author contributions

All authors contributed to the study conception and design. DM, LZ and KX initiated the project. QA and DM drafted and wrote the manuscript. JZ, ZW, YS, BY, and XZ collected the references. All authors contributed to the article and approved the submitted version.

## Funding

This work was supported by the National Natural Science Foundation of China [grant number 82001741] and the Applied Basic Research Project of the Shanxi Science and Technology Department [grant number 201801D221380]

## Conflict of interest

The authors declare that the research was conducted in the absence of any commercial or financial relationships that could be construed as a potential conflict of interest.

## Publisher’s note

All claims expressed in this article are solely those of the authors and do not necessarily represent those of their affiliated organizations, or those of the publisher, the editors and the reviewers. Any product that may be evaluated in this article, or claim that may be made by its manufacturer, is not guaranteed or endorsed by the publisher.
